# NCR1 Expression Identifies Canine Natural Killer Cell Subsets with Phenotypic Similarity to Human Natural Killer Cells

**DOI:** 10.3389/fimmu.2016.00521

**Published:** 2016-11-23

**Authors:** Jennifer A. Foltz, Srinivas S. Somanchi, Yanwen Yang, Arianexys Aquino-Lopez, Erin E. Bishop, Dean A. Lee

**Affiliations:** ^1^Department of Pediatrics-Research, MD Anderson Cancer Center, The University of Texas, Houston, TX, USA; ^2^Health Science Center, Graduate School of Biomedical Sciences, The University of Texas, Houston, TX, USA; ^3^The University of Notre Dame, Notre Dame, IN, USA

**Keywords:** natural killer, osteosarcoma, immunotherapy, NKp46, NCR1, canine, comparative oncology

## Abstract

Canines spontaneously develop many cancers similar to humans – including osteosarcoma, leukemia, and lymphoma – offering the opportunity to study immune therapies in a genetically heterogeneous and immunocompetent environment. However, a lack of antibodies recognizing canine NK cell markers has resulted in suboptimal characterization and unknown purity of NK cell products, hindering the development of canine models of NK cell adoptive immunotherapy. To this end, we generated a novel antibody to canine NCR1 (NKp46), the putative species-wide marker of NK cells, enabling purification of NK cells for further characterization. We demonstrate that CD3^−^/NKp46^+^ cells in healthy and osteosarcoma-bearing canines have phenotypic similarity to human CD3^−^/NKp46^+^ NK cells, expressing mRNA for CD16 and the natural cytotoxicity receptors NKp30, NKp44, and NKp80. Functionally, we demonstrate with the calcein release assay that canine CD3^−^/NKp46^+^ cells kill canine tumor cell lines without prior sensitization and secrete IFN-γ, TNF-α, IL-8, IL-10, and granulocyte-macrophage colony-stimulating factor as measured by Luminex. Similar to human NK cells, CD3^−^/NKp46^+^ cells expand rapidly on feeder cells expressing 4-1BBL and membrane-bound IL-21 (median = 20,283-fold in 21 days). Furthermore, we identify a minor Null population (CD3^−^/CD21^−^/CD14^−^/NKp46^−^) with reduced cytotoxicity against osteosarcoma cells, but similar cytokine secretion as CD3^−^/NKp46^+^ cells. Null cells in canines and humans have reduced expression of NKG2D, NKp44, and CD16 compared to NKp46^+^ NK cells and can be induced to express NKp46 with further expansion on feeder cells. In conclusion, we have identified and characterized canine NK cells, including an NKp46^−^ subset of canine and human NK cells, using a novel anti-canine NKp46 antibody, and report robust *ex vivo* expansion of canine NK cells sufficient for adoptive immunotherapy.

## Introduction

Canines are a large animal model with spontaneous development of many cancers, including osteosarcoma, leukemia, lymphoma, glioblastoma, prostate cancer, and mammary cancer. Canines provide an outbred, immune competent disease model with genetic heterogeneity and a shared environment with humans (1–4). Testing of novel therapies in the canine model has educated the protocols for bone marrow transplants in humans and, more recently, has been used for the testing of immune therapies such as adoptive transfer of T-cells, HER2-*Listeria* vaccine, and Liposomal-muramyl tripeptide (L-MTP-PE; mifamurtide) (5–12).

Despite the advantages of the canine model, NK cells are less well characterized in canines than mice and humans. The sequencing of the canine genome in the early 2000s revealed that like humans, canines have all of the natural cytotoxicity receptors along with NKp80 in their genome (13–17). The primary inhibitory receptors that mediate licensing of NK cells are the Ly49 and KIR families of receptors, both of which recognize self through binding to MHC Class I. Mice have 16 Ly49 genes but only 2 KIR, whereas humans have 16 KIR genes but only a pseudogene of the Ly49 family (18). The canine genome has no KIR and only one Ly49 gene, which has a predicted ITIM sequence suggesting that it functions as an inhibitory receptor (19).

The identification of NK cells in canines has been met with seemingly conflicting results with some studies reporting CD3^−^ cell populations with NK cell properties, while others report CD3^+^ cell populations with NK cell properties (20–23). Recently, Grondahl-Rosado et al. provided more clarity on the phenotype of canine NK cells using a cross-reacting anti-bovine antibody to NCR1 (NKp46), the putative species-wide marker of NK cells in mammals (13–16, 24–27). Using this antibody, they identified a CD3^−^/NKp46^+^ cell population in most canines that were also positive for Granzyme B. Furthermore, they confirmed that NKp46 is an activating receptor in canine. They also proposed that a CD3^−^/NKp46^−^/Granzyme B^+^ cell subset may be a subset of canine NK cells (16, 17). However, this anti-bovine NKp46 antibody is reported by the authors to not be suitable for sorting of CD3^−^/NKp46^+^ cells, limiting the ability to further characterize the receptor expression and function of CD3^−^/NKp46^+^ cells and this NKp46^−^ cell population (16, 17). Additionally, expansion of canine NK-like cells, while more successful than *ex vivo* expansion of mouse NK cells, has been significantly less than reported in humans with expansions reported of up to 233-fold on average in 2–3 weeks (19–23, 28, 29).

We sought to further characterize canine NK cells for use in osteosarcoma, where survival for metastatic human OS patients has largely remained stagnant at only 30% 5-year survival rate for the last 30 years (30–33). Canine OS is highly prevalent, with over 8,000 new diagnoses per year, and an average survival rate of only 1 year, allowing for the rapid testing of new therapeutics. While mouse models have provided important discoveries in OS pathogenesis and treatment, the spontaneous canine model of OS has been well characterized and is used as an additional important animal model of OS (1, 2, 34, 35).

To this end, we defined canine NK cells by their expression of NKp46, using a novel anti-canine NKp46 antibody, and expanded canine NK cells on membrane-bound IL-21 expressing feeder cells. We report here the identification and characterization of NKp46^+^ and NKp46^−^ canine NK cells that have striking phenotypic and functional similarity to human NK cells. Canine NK cells from both healthy and OS-bearing canines expand 20,283-fold in 3 weeks enabling their use in testing NK cell therapies in the spontaneous canine model of OS.

## Materials and Methods

### Peripheral Blood Mononuclear Cell Isolation

Animal research was conducted with approval from the Institutional Animal Care and Use Committee at MD Anderson Cancer Center (00001532-RN00). Healthy canine blood was obtained from established animal colonies at the following locations: Animal Blood Resources International, Hemopet, and Texas A&M University (IACUC Protocol: 2014-0294). Healthy canine blood from UC Davis was exempted from IACUC approval. All blood from client-owned animals was obtained with informed consent and was consistent with the established guidelines for safe canine blood draws. Blood from canine patients with suspected osteosarcoma who had not received chemotherapy within 1 month was obtained with informed consent from UC Davis under Protocol 18315. Osteosarcoma diagnosis was confirmed with radiographs or biopsies. Two out of the seven canine patients presented with potential chondroblastic osteosarcoma, with one of the two patients presenting with disease with alternative diagnosis of chondrosarcoma. Four out of the seven patients were females with ages ranging from 2 to 13 years old. Osteosarcoma tumor sites were frontal bone (1), metatarsal (1), tibia (1), femur (1), radius (2), and ileal body (1). Experiments using discarded buffy coats from normal human red blood cell (RBC) donations were conducted under MD Anderson Cancer Center IRB exemption PA13-0978.

Canine blood was drawn into lithium heparin tubes and was diluted upon receipt 1:5 in HBSS before Ficoll separation. Both canine and human blood were processed using Ficoll Plus (GE Healthcare; 17-1440-02), as described previously, and canine RBCs were lysed with RBC lysis buffer (Stem Cell Technologies, 07800) for 4 min on ice (36, 37).

### Cell Culture

The canine cell lines OSCA8 (OS of the humerus, Jaime Modiano, University of Minnesota) and OSCA40 (OS of the femur, Jaime Modiano,), Gray (OS lung metastasis, University of Wisconsin, Greg MacEwen), Abrams (OS, Ryan Roberts, Research Institute at Nationwide Children’s Hospital), OSCA-78 (femur OS, Kerafast, EMN004), and melanoma-12 (Carlos Rodriguez, UC Davis) were cultured in DMEM High Glucose (Hyclone, SH30243.FS) with 10% FBS (Hyclone, SH30071.03HI), Penicillin Streptomycin Amphotericin Antibiotics (Lonza, 17-745E), and HEPES (OSCA-78 only; Gibco, 15630-080). Canine thyroid adenocarcinoma (CTAC) (Ohio State University, George Krakowka) was cultured in RPMI 1640 (Hyclone, SH30096.FS) with 10% FBS, Glutamax (ThermoFisher Scientific, 35050061), and Antibiotics. Manin–Darby canine kidney cells (MDCK) (Sigma-Aldrich, 84121903-1VL) were cultured in Eagles Minimum Essential Media (Hyclone, SH30244.FS) with 10% FBS, PSA, Glutamax, and 1% Minimum Essential Amino Acids (Sigma, M7145). All cell lines reported in this study were identified as canine origin by species-specific PCR and were unique from each other by canine STR fingerprinting (CellCheck Canine, IDEXX BioResearch) (Table 1). Only MDCK had an established genetic profile with which to compare, which matched the results obtained for our MDCK cell line. The OSCA8 cell line was reported as having been derived from a male, but fingerprinting of our OSCA8 cell line showed only chromosome X (38). This could be caused by a loss of chromosome Y during culture over time. Cells were dissociated for cytotoxicity assays using enzyme-free Cell Dissociation Buffer, Hank’s Based (ThermoFisher Scientific, 13150016). NK cells were cultured in RPMI 1640 media plus Glutamax, 10% FBS, and antibiotics. All cells were routinely tested for *Mycoplasma* contamination using Lonza MycoAlert (Lonza, LT027-58) and found to be negative at all time points with the exception of melanoma-12. K562 feeder cells were derived by transducing K562, a chronic myelogenous leukemia cell line, with human 4-1BBL and membrane-bound human IL-21 (Clone9.mbIL21), as described previously (36).

**Table 1:** **STR profile for canine cell lines used in this paper**.

Marker name	Locus	Chromosome	MDCK	OSCA78	Gray	OSCA8	OSCA40	Abrams	Melanoma-12	CTAC
Canine 1	FH3210	2	239, 305	285	281, 301	281, 285	241, 289	289, 309	297, 301	241
Canine 17	PEZ12	3	263, 297	271	271	294	260	271, 301	267, 271	275
Canine 2	FH3241	8	252, 264	264	256, 264	256, 264	260	260	260, 264	264
Canine 3	FH2004	11	170, 178	174	174	174	174	174	178, 222	243
Canine 16	FH2054	12	152, 172	160, 164	156	156	156, 160	160, 177	165, 169	165
Canine 4	FH2658	14	108, 116, 120	116, 128	109, 120	112	116	105, 120	116	116
Canine 5	FH4012	15	131, 135	131, 135, 140	123, 127	119, 123	127, 140	135	123, 127	123
Canine 6	REN214L11	16	154	154	150	154	154	150	155	154
Canine 13	WILMS-TF	18	275	295, 299	280, 290	283	295	291, 295	291	283, 288
Canine 7	FH2010	24	159	163	151, 159	151	155, 159	151, 159	155	151, 155
Canine 14	PEZ6	27	181, 187	180	180, 184	180	172, 180	180, 187	184	184
Canine 19	VWF.X	27	155	155	179	179	155, 174	161, 179	173	161
Canine 8	FH2361	33	244, 246	NP	248, 252	240, 252	244, 260	244	244, 248	244, 252
Canine 15	FH2611	36	200, 212	208	197	197	201	197, 212	197, 212	204
Sex			X, X	X, Y	X, X	X, X	X, X	X, X	X, Y	X, X

*NP, no product*.

### NK Cell Expansion

Five million canine PBMC were cocultured with 10^6^ K562 Clone9.mbIL-21 in at day 0 (1:2 ratio), and additional K562s were added at a 1:1 ratio at days 7 and 14. Medium was supplemented with 6.1 ng/mL recombinant canine IL-2 (rcIL-2) (R&D systems, 1815-CL), and fresh medium was added every 2–3 days. For human IL-2 comparison expansions, human IL-2 was added at 100 IU/mL in place of canine IL-2. The concentration of human IL-2 was based on the dose of IL-2 used in previous publications on canine NK-like cells (21, 22, 39).

For CD3 depletion experiments, canine PBMC was stained with CD3-FITC antibody and sorted for all PBMC that were CD3-negative. Sodium azide in CD3 antibody was reduced using Amicon Ultra-0.5 mL Centrifugal Filter Units with Ultracel-50 Membrane (EMD Millipore, UFC505024). Expansion was done according to the previous protocol, comparing CD3-depleted PBMC to -undepleted PBMC. Human NK cells were isolated *via* RosetteSep Human NK Cell Enrichment Cocktail at day 0 (Stem Cell Technologies; 15065) and expanded with K562 mbIL-21 feeder cells with the same protocol using 50 IU/mL of human IL-2.

Fold expansion was calculated as the percentage of CD3^−^/NKp46^+^ cells within the lymphocyte gate of total PBMC. Null cell calculations were determined by subtracting the percentage of CD21 and CD14 expressing cells in the lymphocyte gate from the CD3^−^ cell percentage.

### Sequencing

The sequence of canine NKp46 was verified with the following primers: Forward: 5′ ACTCACTGCCCTTCTCTTCC 3′, Reverse: 5′ CTAACTGTGGCCAGCACATC 3′ *via* RT-PCR with the following conditions Initial Denaturation: 94°C – 3 min, Denature: 94°C – 30 s, Annealing: 58°C – 30 s, Extension: 68°C – 1 min, Final Extension: 68°C – 5 min for 35 cycles using Platinum Taq DNA Polymerase High Fidelity (ThermoFisher Scientific, Cat: 11304-011) and dNTPs (ThermoFisher Scientific, Cat: 18427013). The PCR product was cloned into pCR2.1 Vector using TA cloning (ThermoFisher Scientific, K202020) and sequenced using Sanger-based sequencing on an ABI 3730 with M13 (-21) Forward and M13 Reverse Primers.

### NKp46 Antibody Production

A mouse monoclonal antibody specific to canine NKp46 was constructed by first confirming the expression and sequence of NKp46 in expanded canine lineage negative (CD3^−^/TCR^−^/CD21^−^/CD14^−^) cells, using primers which covered 940/955 bp of canine NKp46 (*NM_001284448.1*). The sequence obtained matched the NCBI database sequence (data not shown). A chimeric protein composed of the signal sequence of human granulocyte-macrophage colony-stimulating factor (GM-CSF, NP_000749.2; aa: 1–17), the extracellular domain of canine NKp46 as determined using Phobius protein prediction software (NP_001271377.1; aa: 21–255), and murine CD8α extracellular and transmembrane regions (NP_001074579.1; aa: 28–247) was constructed and synthesized using Life Technologies (custom order). This construct was ligated into the MigR1 vector (Provided by Patrick Zweidler-McKay), and virus was produced using GP2-293 packaging cells (Clontech, 631530) with 0.8 μg pVSV-G packaging vector (Clontech), 10 μL Lipofectamine 2000, and 4 μg of the NKp46-MigR1 vector or Empty vector per well of a six-well plate. Supernatant was collected after 40 h, spun down, and added to L-cells (ATCC, CRL-2648) along with 8 μg/mL polybrene. Cell surface expression of the chimeric NKp46 protein was verified through flow staining with anti-mouse CD8α antibody (BD Biosciences; 561093) and GFP. BALB/C mice were immunized *via* footpad with L-cells expressing NKp46 fusion protein for 33 days (NKp46:L-cells), as described previously (40).

### Flow Cytometry

Blocking was performed in blocking buffer (50% FBS/PBS) with 0.1 mg of dog gamma globulin (Jackson ImmunoResearch, 0004-000-002) per 1,000,000 cells. Cells were stained with 200 ng/1,000,000 cells of mouse anti-canine NKp46 Clone 48A (isotype: IgG2a) or unconjugated TCRαβ/TCRγδ for 30 min, followed by goat anti-mouse IgG RPE (Jackson ImmunoResearch, 115-116-146) in 50% FBS/PBS, and finally with anti-canine TCRαβ/γδ (FITC/Alexa Fluor 488 when indicated; Peter Moore, UC Davis) and commercially available antibodies (Biorad) to canine CD3 (MCA1774F; clone CA17.2A12), CD8α (MCA1039A700; clone YCATE55.9), CD4 (MCA1038PECY7; clone YKIX302.9), CD21 (MCA1781A647; clone CA2.1D6), CD5 (MCA1037APC; YKIX322.3), and anti-human CD14 (MCA1568A647, MCA1568A700; TÜK4). Cells events were acquired on a LSR Fortessa. Flow cytometry gating was determined using cells stained with secondary only and single color controls were analyzed using FlowJo 7.6.5/10.

### Cell Sorting

Canine cells were sorted on a FACSAria Sorter at the flow cytometry core facility (UT MD Anderson Cancer Center) using four-way purity with post-sort purity verification for expression of CD3 and NKp46 to generate ≥99.5% pure populations of CD3^−^/NKp46^+^, CD3^−^/NKp46^−^, CD3^+^, and CD3^−^ cells when indicated.

### Luminex

Luminex assays were done, as described previously, using tissue culture supernatant from 2 × 10^5^ sorted, CD3^−^/NKp46^+^, CD3^−^/NKp46^−^, or CD3^+^ expanded cells cocultured for 6 h at 37°C with rcIL-2 or rcIL-2 plus 4 × 10^5^ K562 Clone9.mbIL-21 irradiated feeder cells with the CCYTOMAG-90K Canine Cytokine Chemokine Panel (EMD Millipore, CCYTMG-90K-PX13) on a Bioplex-200 using Bioplex Manager 6.0 Software (Biorad, USA) (41). Cytokine secretion with coculture with K562 Clone9.mbIL-21 feeder cells was normalized by subtracting cytokine secretion in IL-2 only.

### Cytotoxicity Assays

Canine CD3^−^/NKp46^+^ were sorted when necessary to be ≥95% pure. Comparison of CD3^−^/NKp46^+^, CD3^−^/NKp46^−^, and CD3^+^ expanded cells were sorted as described, rested overnight in IL-2, and cytotoxicity assays with calcein-AM based method were conducted in at least duplicate using 4 μg of calcein-AM/1,000,000 target cells (37).

### RT-PCR/qPCR

RNA was isolated from canine expanded cells using RNAeasy Kit, QiaShredder Columns, and RNAase-Free DNase Set (all Qiagen, 74104, 79654, 79254), and cDNA was synthesized using Omniscript RT Kit (Qiagen, 20511) with RNase inhibitor (New England Biolabs, M0307S) and Oligo(dt)_20_ (ThermoFisher Scientific, 18418-020). RT-PCR and qPCR reactions were performed on Roche 480 (Roche, USA) using Power Up Sybr Green Master Mix (ThermoFisher; A25742) or TaqMan Universal PCR Mastermix for qPCR for CD16 (ThermoFisher Scientific, 4304437) for 40 cycles. All qPCR was done in duplicate with at least three different donors. Semi-quantitative RT-PCR reactions were run on five different donors with 100 ng cDNA per sample and 6× DNA loading dye, and products were verified on a 1.5% agarose gel with TAE buffer and GelRed Nucleic Acid Stain (Phenix, RGB-4103) and 100 bp DNA Ladder (New England Biolabs, N3238S). Gels were imaged on BioRad ChemiDoc Touch Imaging System (BioRad, USA) for Faint Bands and analyzed using Image Lab 5.2.1. The following enhancements were uniformly applied to all gels (High: 65535, Low: 34, Gamma: 0.43).

PCR cycling conditions were as followings: UDG activation: 50°C – 2 min, Polymerase Activation: 95°C – 2 min, Denature: 95°C – 15 s, B-actin, NKp46, NKp30, Granzyme B, and Perforin – Anneal/Extend: 62°C – 1 min; B-actin, Ly49, and CD16 – Anneal: 54°C – 15 s, Extension: 72°C – 1 min; B-actin, DNAM-1, NKp30, NKp44, NKG2D, and NKp80 at the same conditions as Ly49 and CD16 with the exception of Annealing at 59°C. Primer sequences for NKp30, NKG2D, Perforin, and Granzyme B were published and verified previously (22). Primers for all other genes were made using Blast Primer software and Primer 3 with predicted specificity verified by Blast, as follows: Ly49: Forward-5′ TGGAAATGGGTTGACACTGA 3′, Reverse-5′ CTTTGGAAGGAGGCAGACAA 3′; NKp46: Forward-5′ CCGCATCCCAAAAATGACCC 3′, Reverse-5′ GGGCCTATGGGAAACTCCAC 3′; NKp80: Forward-5′ TCCCTGTCCTTACTGGTTTCTC 3′, Reverse-5′ TATTTGAGCCATTCCGATG 3′; CD16: Forward-5′ CAGTGGAGAGTACAGGTGCC 3′, Reverse-5′ CCCTGCAGAAGTAGGAGCC 3′; DNAM-1: Forward-5′ AATGTCACGCTCACCTGTCA 3′, Reverse-5′ CGGTAAAGTCCCGAGTCAGA 3′; NKp44: Forward-5′ GAAGGAGGAAGACTCGGGATA 3′, Reverse-5′ GAAGGGGTGATGGTGGTAGA 3′; and B-actin: Forward-5′ CGAGCATCCCCAAAGTTCTA 3′, Reverse-5′ ATTCTCTTTCCCTCCCCTGT 3′.

Primer specificity was verified by Melt Curve Analysis and agarose gel electrophoresis. Primer product sequences for new primers were verified using Qiaquick Gel Extraction Kit (Qiagen, 28704) and sequencing, as described previously. Primers were synthesized by Sigma-Aldrich. For qPCR for CD16, TaqMan Universal PCR Master Mix was used with B-actin (ThermoFisher Scientific, Catalog #: 4453320, Assay ID: Cf03023880_g1) and CD16 probes (ThermoFisher Scientific, Catalog #: 4448892 Assay ID: Cf02645051_m1) for 40 cycles according to the TaqMan Universal PCR Master Mix protocol. qPCR was determined using the $2^{-\text{ΔΔ}{\text{C}}_{t}}$ method and normalized to B-actin, and relative to CD3^−^/NKp46^+^ cells from the same donor.

### Mass Cytometry

Antibodies for mass cytometry were labeled with heavy metals using Maxpar-X8 labeling reagent kits (DVS Sciences) according to manufacturer’s instructions and titrated for determination of optimal concentration. The antibodies and their respective heavy metal labeling include CD314/NKG2D-150Nd (Biolegend, 320802), CD3-151Eu (BioLegend, 300443), CD335/NKp46-154Sm (BioLegend, 331902), CD56-162Dy (BD, 559043), CD336/NKp44-163Dy (BioLegend, 325102), CD337/NKp30-164Dy (BioLegend, 325202) CD16-165Ho (DVS, 3165001B), CD159a/NKG2A-166Er (R&D, MAB1059), CD226/DNAM1-167Er (BioLegend, 337102), and CD159c/NKG2C-169Tm (R&D, MAB1381). Frozen primary and K562 Clone9.mbIL-21 expanded human NK cells were thawed 1 day prior to staining to allow for NK cell recovery overnight. After overnight recovery, 1.5 × 10^6^ cells were stained with 2.5μM cell ID cisplatin (Fluidigm, 201064) in serum-free RPMI for 1 min for identification of viable populations. Subsequently, CyTOF staining was performed, as described previously, with the following minor modification: staining was performed in 5% FBS/0.01% sodium azide buffer in PBS (42). Data were acquired on the CyTOF instrument (DVS Sciences).

### Clustering Analysis

Clustering analysis of mass cytometry data was performed using spanning-tree progression analysis of density-normalized events (SPADE V3.0) software (43). Markers used for clustering of data were CD56, NKp46, NKp30, NKp44, NKG2A, NKG2C, and NKG2D.

### Statistical Analysis

Statistical analysis was performed using GraphPad Prism 6.0. Luminex assays were analyzed with unpaired *t*-tests and Holm–Sidak method for multiple comparisons. Paired *t*-tests were run for the following: cytotoxicity assays between NK and T-cells or Null Cells, qPCR, and comparison of NKp46^+^ and NKp46^−^ human NK cell phenotype. Comparison between healthy and OS-bearing canines’ percent NK cells and expansion used the Mann–Whitney test. All other statistical analyses were unpaired Student’s *t*-tests (all two-tailed). Human versus canine IL-2 analysis used a one-tailed Wilcoxon-matched pairs test. Correlations were done using the Spearman *r* test. *p* Values less than 0.05 were considered significant.

## Results

### Construction of an Anti-Canine NKp46 Antibody

**Figure 1:**
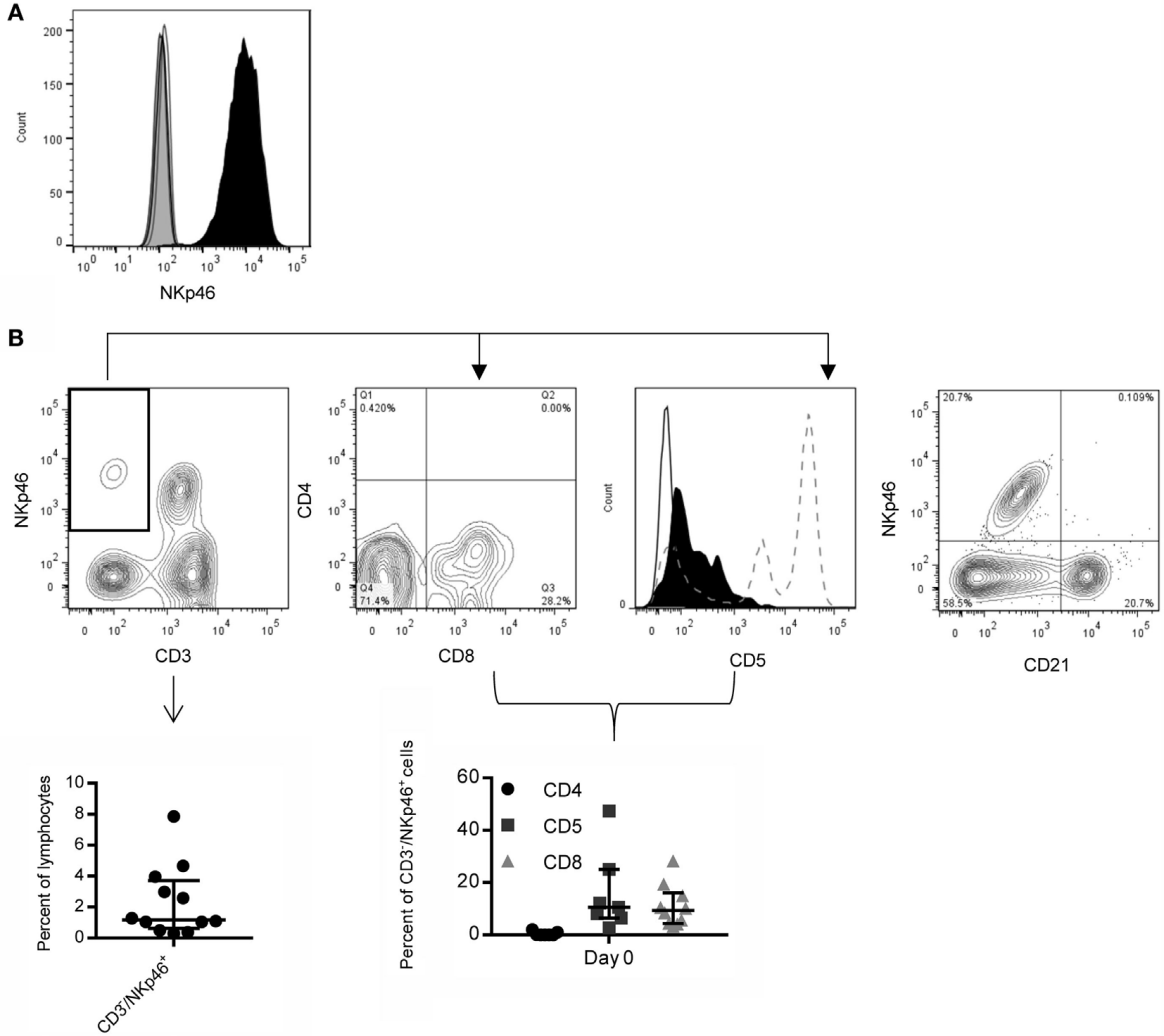
**Expression of NKp46 on canine PBMC using a novel anti-canine NKp46 antibody**. **(A)** Clone 48A is specific for L-cells expressing NKp46 (open histograms: secondary antibody only, gray filled: vector only, black filled: NKp46:L-cells). **(B)** NKp46 is expressed on CD3^−^ and CD3^+^ lymphocytes. CD3^−^/NKp46^+^ cells make up on average 2.3% of lymphocytes. Shown is median ± IQR (*n* = 12). Representative flow plot is shown. These CD3^−^/NKp46^+^ cells are largely negative for CD4 (median = 0%), and a subset express CD8α (median = 9.3%). They are CD5^low/−^ (median = 10.6%) (Solid line: negative control, Black filled: CD3^−^/NKp46^+^, Dotted line: CD5 expression on total lymphocytes showing two populations of CD5^+^ cells). Shown is median ± IQR for CD4 (*n* = 7), CD5 (*n* = 7), and CD8 (*n* = 10). NKp46 is not expressed on CD21^+^ B-cells.

Hybridomas obtained by immunization with L-cells expressing the NKp46 fusion protein (NKp46:L-cells) were screened *via* ELISA and flow cytometry against NKp46:L-cells and Empty Vector:L-cells. Clone 48A had strong staining on NKp46:L-cells and was selected for all future experiments (Figure 1A). Clone 48A did not identify NKp46 by western blot or immunoprecipitation (data not shown).

### NKp46 Expression on Canine Peripheral Blood Mononuclear Cells

CD3^−^/NKp46^+^ cells in PBMC were largely negative for expression of CD4 (median = 0%, IQR = 0, 1.0, *n* = 7), with low expression of CD5 (median = 10.6%, IQR = 6.4, 25, *n* = 7) and CD8 (median = 9.3%, IQR = 4.4, 16.0, *n* = 10). NKp46 was not coexpressed with macrophage (CD14, data not shown) or B-cell lineage markers (CD21, Figure 1B). On average, 2.3% (median = 1.2%, IQR = 0.6, 3.7, *n* = 12) of lymphocytes from healthy canines were CD3^−^/NKp46^+^, consistent with an NK cell expression pattern (Figure 1B). NKp46 was also expressed on a subset of CD3^+^/TCR^+^ cells.

### *Ex Vivo* Expansion of Canine CD3^−^/NKp46^+^ Cells

**Figure 2:**
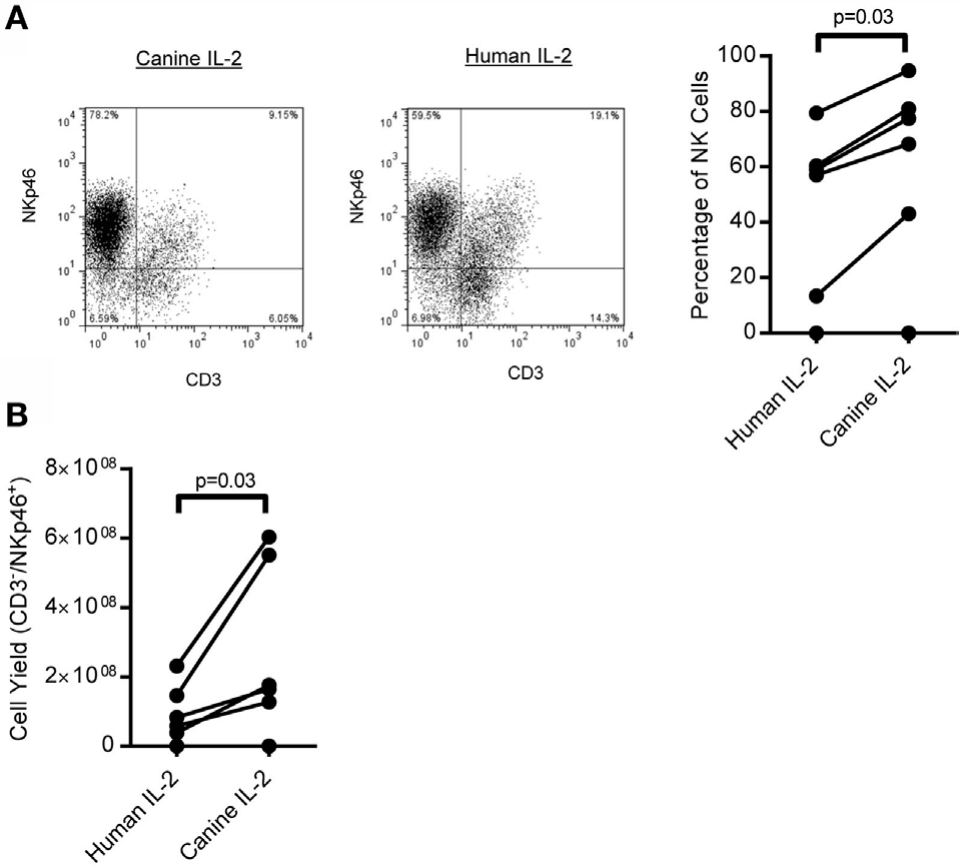
**Expansion of CD3^−^/NKp46^+^ cells on K562 mbIL-21 feeder cells with human versus canine IL-2**. **(A)** Canine CD3^−^/NKp46^+^ cells expanded on K562 Clone 9.mbIL-21 feeder cells with canine IL-2 have significantly increased purity of CD3^−^/NKp46^+^ cells at day 21 compared to expansion with human IL-2 (*n* = 6) representative flow plot shown. Canine donors are depicted by connecting lines. **(B)** CD3^−^/NKp46^+^ cells expanded with canine IL-2 have an increase in yield of CD3^−^/NKp46^+^ cells at day 21.

Next, we expanded canine CD3^−^/NKp46^+^ cells from whole PBMC using K562 Clone9.mbIL-21 feeder cells in a 21-day culture, since we previously demonstrated significantly improved expansion of human NK cells with K562 Clone9.mbIL-21 feeder cells compared to K562 mbIL-15 feeder cells (36). The percentage of CD3^−^/NKp46^+^ cells significantly improved at the end of expansion when using canine IL-2 (rcIL-2) compared to human IL-2 (*p* = 0.03, Human IL-2: median = 58.02, IQR = 10.05, 65.16; Canine IL-2: median = 72.9, IQR = 32.25, 84.35; *n* = 6) (Figure 2A). There was significantly increased yield of CD3^−^/NKp46^+^ cells (*p* = 0.03) at the end of expansion with rcIL-2 (Figure 2B) (Human IL-2: median = 7.07 × 10^7^, IQR = 2.9 × 10^7^, 1.67 × 10^8^; canine IL-2: median = 1.71 × 10^8^, IQR = 9.58 × 10^7^, 5.65 × 10^8^, *n* = 6). For all future expansions reported, rcIL-2 was used.

**Figure 3:**
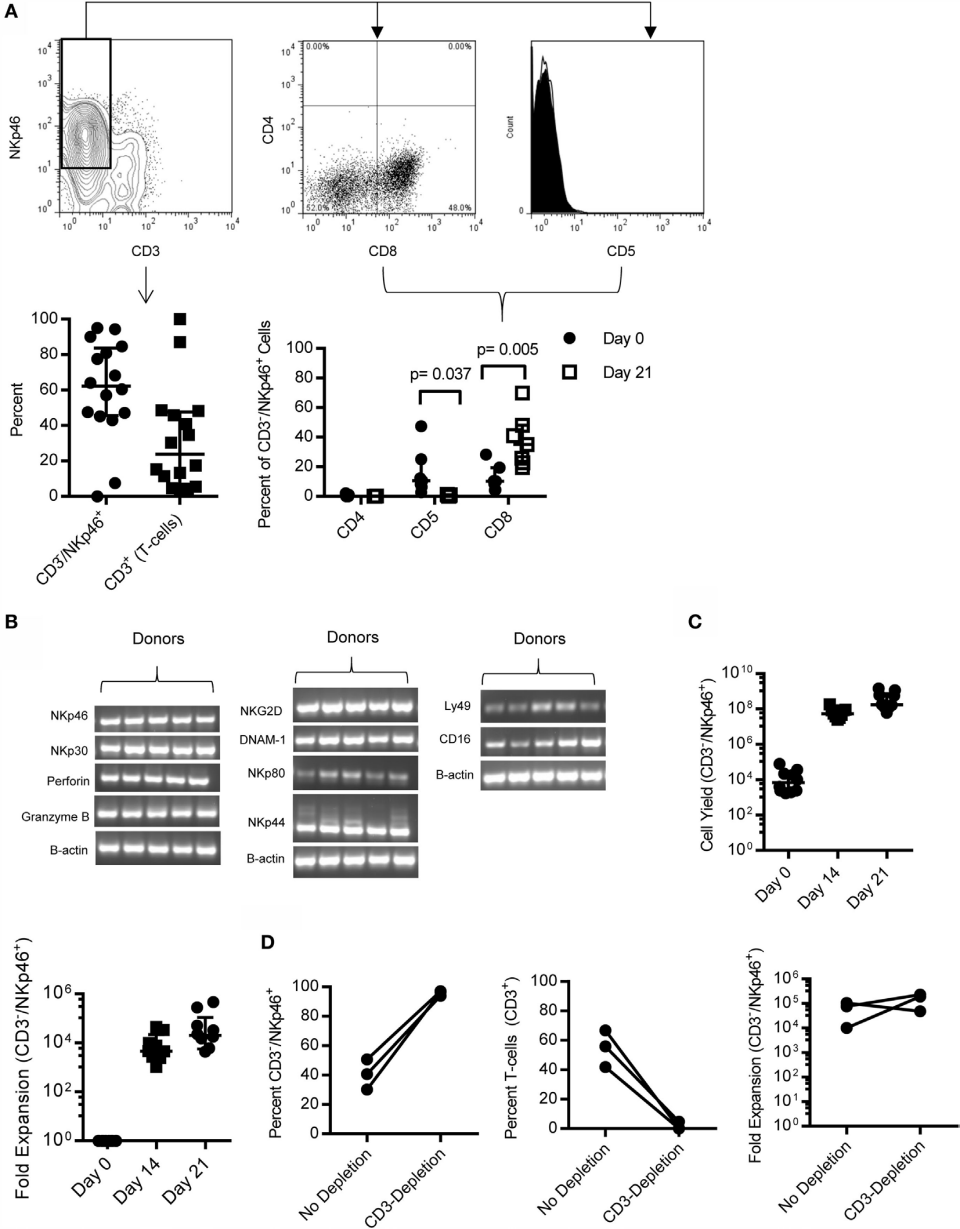
**CD3^−^/NKp46^+^ cells expand rapidly on K562 mbIL-21 feeder cells and express NK-cell associated markers**. **(A)** CD3^−^/NKp46^+^ cells expand on K562mbIL-21 feeder cells to make up on average 60.2% of total cells, with contaminating T-cells on average 31.8% (*n* = 16, median ± IQR shown). Expanded CD3^−^/NKp46^+^ cells are CD4^−^ (median = 0.08%) and are significantly decreased in percentage of cells coexpressing CD5 (median = 0.4%, *p* = 0.037) (open histogram = negative control, black filled = CD3^−^/NKp46^+^) and increased in CD8 coexpression (median = 35%, *p* = 0.005, *n* = 7; median ± IQR shown). **(B)** CD3^−^/NKp46^+^ cells express mRNA for NKp46, NKp30, Perforin, Granzyme B, NKG2D, DNAM-1, NKp80, NKp44, Ly49, and CD16 (*n* = 5, all PCR reactions were conducted concurrently). **(C)** On average, 4.5 × 10^8^ (median = 1.7e8) CD3^−^/NKp46^+^ cells are produced at the end of 3 weeks from an average 17,774 CD3^−^/NKp46^+^ (median = 7,000) cells at day 0 (shown is median ± IQR) representing a median 20,283-fold expansion. **(D)** Depletion of T-cells at day 0 significantly increases the purity of CD3^−^/NKp46^+^ cells at day 21 (95.8 versus 40.6%) and does not hinder fold expansion (157,281 versus 64,686, *n* = 3).

When PBMC was cultured for 21 days on K562 Clone9.mbIL21 with rcIL-2, CD3^−^/NKp46^+^ cells made up a median 62.31% (IQR = 45.7, 83.7, *n* = 16) and CD3^+^ T cells comprised a minor portion (median = 23.85%, IQR = 6.9%, 47.7%, *n* = 16) of the total expanded cell product (Figure 3A), with the exception of two outliers. The phenotype of the CD3^−^/NKp46^+^ cells significantly changed during expansion, with CD5 decreasing (*p* = 0.037, Median = day 0: 10.6, IQR = 6.42, 25; day 21: 0.35, IQR = 0.1, 1.02) and CD8α increasing (*p* = 0.005, Median = day 0: 9.33, IQR = 4.35, 16.03; day 21: 35, IQR = 22.7, 48.1) (Figure 3B). In order to further characterize the expanded CD3^−^/NKp46^+^ cells, for which antibodies to other NK cell receptors are not available, semi-quantitative RT-PCR was performed on sorted CD3^−^/NKp46^+^ for several known human or mouse NK cell-associated genes for which canines are predicted to have homologous genes. Expanded NK cells from all donors expressed mRNA for NKp46, NKp30, NKp44, NKp80, NKG2D, DNAM-1, CD16, Ly49, Granzyme B, and Perforin, confirming that canine CD3^−^/NKp46^+^ cells express typical NK cell-associated genes (Figure 3C).

Expansion of CD3^−^/NKp46^+^ cells with K562 Clone9.mbIL-21 feeder cells for 21 days yielded a median 1.7 × 10^8^ CD3^−^/NKp46^+^ cells (mean = 4.5 × 10^8^, IQR = 1.1 × 10^8^, 7.5 × 10^8^) from a starting product of 7,000 (median) CD3^−^/NKp46^+^ cells (IQR = 2,375, 25,750, *n* = 10) (Figure 3C) representing a median 20,283-fold expansion (mean = 89,151, IQR = 5,655, 108,749, *n* = 10; 2 weeks = 4,622 median fold, 12,319 mean, *n* = 0). Expansion was successful in 9/10 donors tested. The one donor where expansion was not successful had the lowest percentage of CD3^−^/NKp46^+^ of all donors tested. Since expansion of CD3^−^/NKp46^+^ cells from whole PBMC resulted in donor-dependent T-cell contamination that would be detrimental in an allogeneic setting, we depleted T-cells from PBMC using FACS-sorting, leaving B-cells and macrophages/monocytes in the culture. We previously observed with human NK cell expansion that these cells provide support for NK cell expansion without hindering cell purity (36). Using this approach, the purity of CD3^−^/NKp46^+^ cells significantly increased (median = 96.5 versus 40.7%; *p* = 0.01) and fold expansion was not affected with 2/3 donors demonstrating increased fold expansion with T-cell depletion (Figure 3D).

### Function of CD3^−^/NKp46^+^ Cells

**Figure 4:**
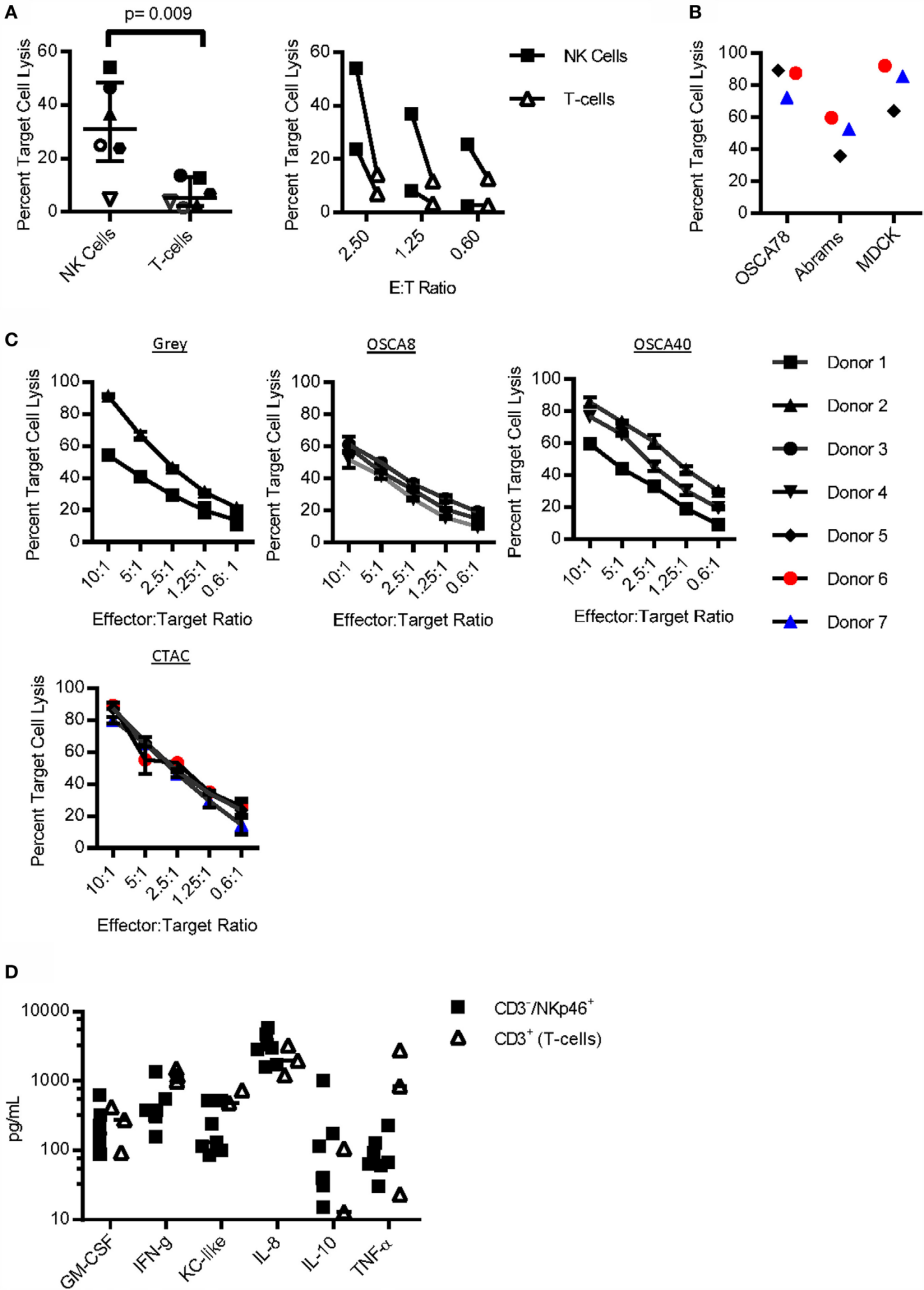
**Function of CD3^−^/NKp46^+^ cells**. **(A)** Sorted CD3^−^/NKp46^+^ cells have increased cytotoxicity against the canine osteosarcoma (Gray) cell line when compared to CD3^+^ T-cells (*n* = 6). **(B)** CD3^−^/NKp46^+^ cells uniformly kill OSCA78, Abrams, and MDCK. **(C)** Canine CD3^−^/NKp46^+^ cells have titratable, donor-dependent cytotoxicity against four different canine osteosarcoma cell lines: Gray (*n* = 2), OSCA8 (*n* = 3), and OSCA40 (*n* = 3), and the canine thyroid adenocarcinoma cell line (CTAC, *n* = 3) (mean ± SD for each donor depicted). **(D)** Expanded CD3^−^/NKp46^+^ cells secrete GM-CSF, IFN-γ, KC-like, IL-8, IL-10, and TNF-α upon coculture with K562 Clone9.mbIL-21 feeder cells (*n* = 7, median depicted). When compared to CD3^+^ T-cells (*n* = 3) from the same donor and also expanded on K562 mbIL-21, canine CD3^−^/NKp46^+^ cells secrete similar cytokines at similar concentrations.

To determine whether CD3^−^/NKp46^+^ cells are able to kill spontaneously, a hallmark of NK cell function expanded pure CD3^−^/NKp46^+^ cells and CD3^+^ T-cells from the same donor were cultured with the canine osteosarcoma cell line, Gray. CD3^−^/NKp46^+^ cells had significantly increased cytotoxicity than T-cells [*p* = 0.009, Median: NK = 30.92, IQR = 19.1, 48.4; T-cells = 5.36, IQR = 2.3, 13.06; 2.5 Effector:Target (E:T) Ratio], and this increase was consistent across three different E:T ratios (Figure 4A). Next, we compared the cytotoxicity of CD3^−^/NKp46^+^ cells against two additional canine osteosarcoma cell lines (OSCA78 and Abrams), along with MDCK as a normal control at a 10:1 E:T ratio (44). We found OSCA78, Abrams, and MDCK to be sensitive to NK cell killing by all three donors (Figure 4B). CD3^−^/NKp46^+^ cells displayed titratable, donor-dependent cytotoxicity against three different canine osteosarcoma cell lines, including metastatic (Gray) osteosarcoma. Furthermore, CD3^−^/NKp46^+^ cells are highly cytotoxic against CTAC (Figure 4C). Thus, CD3^−^/NKp46^+^ cells appear to possess NK cell function and CD3^−^/NKp46^+^ will be used interchangeably with NK cells for the rest of the manuscript.

To determine cytokine secretion capabilities of canine NK cells, NK cells were compared to CD3^+^ T-cells from the same donor after coculture with K562 Clone 9.mbIL-21 feeder cells. Cytokine secretion was similar for all (Figure 4D). There was no detectable secretion of IL-2, IL-6, IL-7, IL-15, IL-18, IP-10, or MCP-1 at baseline or after coculture with K562 Clone 9.mbIL-21 cells in either CD3^−^/NKp46^+^ cells or T-cells (not shown).

### NKp46 Identifies Distinct Subsets of NK Cells

**Figure 5:**
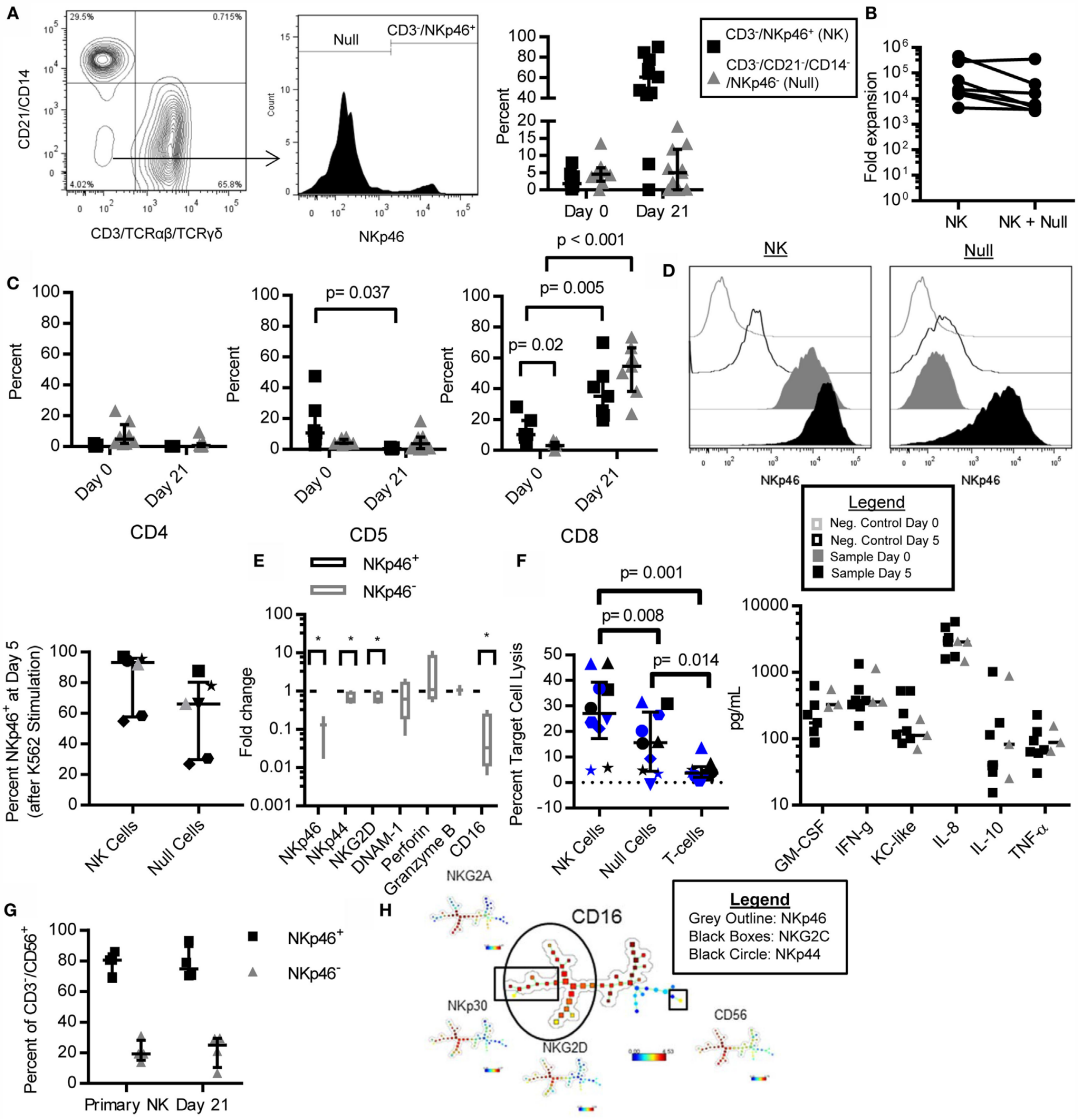

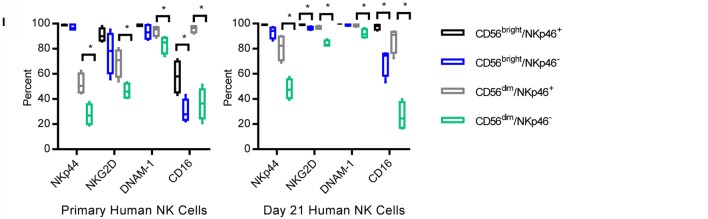
**NKp46^−^ NK cells in canines and humans are CD16^low/−^, retain NK cell phenotype and function, and NKp46 is inducible**. CD3^−^/CD21^−^/CD14^−^, NKp46^−^ (Null) cells are present in canine PBMC **(A)** as described for expanded cells in Figure 3A. Null cells consist of on average 5.1% of lymphocytes in PBMC, and 5.7% of expanded cells (median ± IQR shown, day 0 *n* = 8, day 21 *n* = 12). **(B)** The addition of null cells to fold expansion calculation decreases mean fold expansion by 50% (*n* = 8, median ± IQR shown). **(C)** Null cells have negligible expression of CD4, CD5, and CD8 in PBMC, but increase CD8 expression with expansion similar to CD3^−^/NKp46^+^ cells (3.1–52.4%, *n* = 7, median ± IQR shown). **(D)** NKp46 can be induced in Null cells after 5 days coculture with K562 Clone 9.mbIL-21 feeder cells, while NKp46 expression is constant in sorted CD3^−^/NKp46^+^ cells (*n* = 7). **(E)** Expanded Null cells express similar levels of DNAM-1, Perforin, and Granzyme B, but decreased expression of NKp46, NKG2D, NKp44, and CD16, compared to CD3^−^/NKp46^+^ cells (mean ± SD shown). **(F)** Expanded Null cells have decreased cytotoxicity against Gray and melanoma-12 [*n* = 9, mel-12 (in black) = 4, Gray = 6 (in blue), individual donors designated by unique symbols, median ± IQR shown], but similar cytokine secretion (*n* = 3) compared to CD3^−^/NKp46^+^ cells (*n* = 7, median depicted). **(G)** NKp46^−^ NK cells are present in primary (mean = 21%) and K562 Clone 9.mbIL-21-expanded (mean = 21.7%) human NK cells (*n* = 4, median ± IQR shown). **(H)** Spade clustering analysis of primary and mbIL-21 expanded human NK cells for CD56, NKp46, NKp30, NKp44, NKG2C, NKG2D, and NKG2A revealed that NKp46 and CD16 cluster together (outlining delineates cells that stain positive for the indicated marker) and **(I)** NKp46^−^ cells have decreased expression of CD16, NKp44, DNAM-1, and NKG2D (*n* = 4, median ± IQR shown, whiskers are minimum to maximum).

We observed that NKp46 was not expressed on all lineage negative (CD3^−^/CD21^−^/CD14^−^) cells in both PBMC (Figure 5A) and expanded (Figure 3A) cells, with donor-dependent variation in the percent of null cells (CD3^−^/CD21^−^/CD14^−^/NKp46^−^) observed. Null cells made up 0–13.52% of lymphocytes at day 0 (median = 4.5%, IQR = 2.54, 6.40), and 0–18.4% of the final expanded cell product (median = 5.0%, IQR = 0, 11.8). A significant negative correlation between percent of CD3^−^/NKp46^+^ cells at day 0 and CD3^−^/NKp46^+^ fold expansion was observed (*r* = −0.81, *p* = 0.022, not shown), which led us to question whether Null cells were significantly contributing to the yield of NK cells at day 21. When null cells were included in the fold expansion calculations, the mean fold expansion was 50% less than when using NKp46^+^ cells only, representing at most a two-fold over-estimation of expansion rates if all of the Null cells represented an NK population (Figure 5B).

Next, we determined the phenotype of these Null cells. Null cells had similar expression of CD4 (PBMC: median = 4.7%, IQR = 2.01, 14.2, *n* = 8; day 21: 0.6%, IQR = 0.03, 1.91, *n* = 9) and CD5 (PBMC: median = 4.07%, IQR = 3.72, 6.45, *n* = 8; day 21: 3.5%, IQR = 0.38, 7.89, *n* = 9) compared to CD3^−^/NKp46^+^ (NK) cells, but low expression of CD8 at day 0 (*p* = 0.02, Median = 3.1%, IQR = 0.9, 5.33, *n* = 7). However, similar to NK cells, CD8 expression in the null cells was significantly increased at day 21 (*p* < 0.001, day 21 = 54.6%) (Figure 5C). We questioned whether NKp46 might be inducible in these null cells as has been described in porcine NKp46^−^ cells (27). To address this question, we sorted the expanded cells into pure NK and Null cell populations and restimulated them on K562 Clone 9.mbIL-21 feeder cells for five additional days. At the end of 5 days, NKp46 was expressed in Null cells (Null: median = 66.15, IQR = 29.69, 80.33; NK: 93.32, IQR = 57.57, 96.7) (Figure 5D). Since NKp46 could be induced in formerly Null cells, we then determined if the Null cells express other NK cell-related genes. Since antibodies to other NK cell receptors are not available, we assessed the expression of several key NK cell receptors by qPCR. Confirming our flow cytometry data, NKp46 mRNA was significantly decreased in Null cells (*p* = 0.004). Null cells express other NK cell-related genes with similar levels of Granzyme B (*n* = 3), Perforin (*n* = 4), and DNAM-1 (*n* = 4). However, NKp44 (*p* = 0.04), NKG2D (*p* = 0.04), and CD16 (*p* = 0.009, all *n* = 4) were significantly decreased compared to NK cells (Figure 5E).

Since Null cells are capable of expressing NKp46 along with other NK cell genes, we wanted to determine if Null cells have NK cell function. Expanded NK (CD3^−^/NKp46^+^), Null (CD3^−^/NKp46^−^), and T-cells (CD3^+^) were sorted from the same donor. Null cells had significantly decreased cytotoxicity compared to NK cells (*p* = 0.008), but increased cytotoxicity compared to T-cells (*p* = 0.01) (Median: NK = 27.1%, IQR = 17.3, 39.3; Null = 15.7%, IQR = 4.4, 27.5; T-cell = 3.7%, IQR = 2.07, 6.24). Null cells had similar cytokine secretion capacity compared to NK cells (Figure 5F).

Finally, we sought to determine if human NK cells have similar subsets of NKp46 expressing cells. Using mass cytometry data of human CD3^−^/CD56^+^ primary NK cells and K562 Clone9.mbIL-21-expanded NK cells, we found several striking similarities between human and canine NK cells. NKp46^−^/CD3^−^/CD56^+^ make up 21% of primary NK cells and 21.7% of mbIL-21 expanded NK cells (Figure 5G). NKp46^−^ NK cells are primarily found within the CD56^dim^ NK cell subset (data not shown). Next, we used Spade to determine if NKp46^−^ NK cells are a distinct population of human NK cells. We clustered for CD56, NKp46, NKp30, NKp44, NKG2A, NKG2C, and NKG2D on both primary and mbIL-21 expanded human NK cells and found that NKp46 expression clusters into distinct NKp46^+^ and NKp46^−^ NK populations and NKp46 expression overlaps with CD16 expression (Figure 5H). Primary and expanded human NKp46^−^ NK cells had reduced expression of NKp44 (Primary – CD56^dim^: *p* = 0.0006; Expanded – CD56^dim^: *p* = 0.0004), NKG2D (Primary – CD56^dim^: *p* = 0.02; Expanded – CD56^bright^: *p* = 0.02, CD56^dim^: *p* = 0.002), and CD16 (Primary – CD56^bright^: *p* = 0.0035, CD56^dim^: *p* = 0.0025; Expanded – CD56^bright^: *p* = 0.02, CD56^dim^: 0.0012) (Figure 5I). In contrast to canine Null cells, NKp46^−^ primary and expanded human NK cells had reduced expression of DNAM-1 (Primary – CD56^dim^: *p* = 0.01, Expanded – CD56^dim^: *p* = 0.01) (Figures 5E,I).

### NK Cells in Canine Osteosarcoma

**Figure 6:**
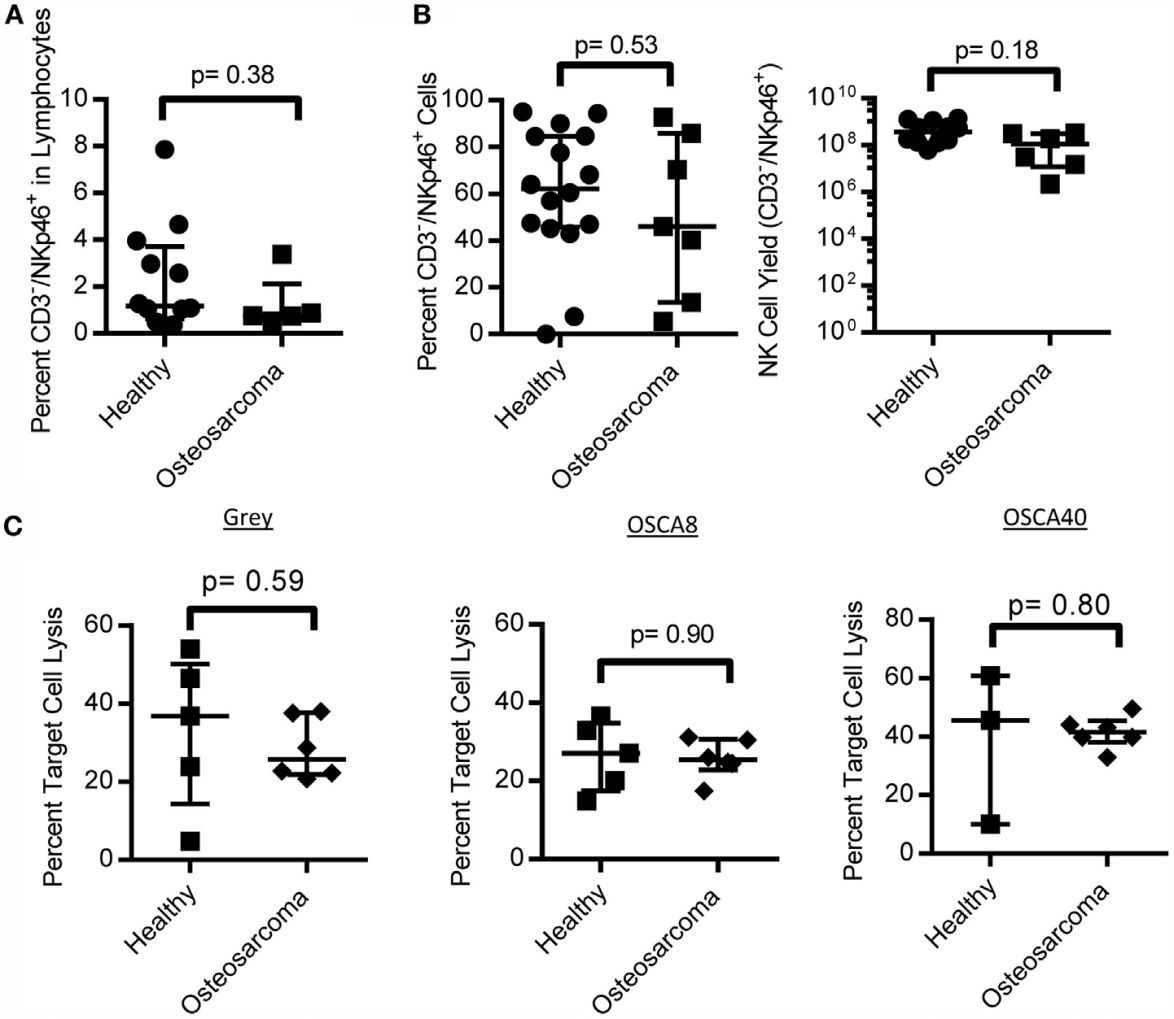
**NK cell numbers, expansion, and function in canine osteosarcoma patients**. Canine osteosarcoma patients have similar percentages of NK cells in lymphocytes **(A)**, retain proliferative capacity in response to K562 Clone9.mbIL-21 feeder cells **(B)**, and cytotoxicity against canine osteosarcoma cell lines when compared to healthy canines **(C)** (median ± IQR for all).

We obtained PBMC from canines that were not currently undergoing chemotherapy and found no difference in the percent of NK cells in OS-bearing canines compared to healthy controls (Figure 6A). Additionally, NK cells from OS patients had similar proliferation on K562 Clone 9.mbIL-21 feeder cells and cytotoxicity against three canine OS cell lines compared to healthy canines (Figures 6B,C).

## Discussion

We sought to characterize canine NK cells and optimize their expansion for use as a comparative oncology model of NK immunotherapy of OS. To this end, we developed a monoclonal antibody specific to canine NKp46 and demonstrated that this antibody recognizes CD3^−^/NKp46^+^ cells that have striking phenotypic and functional similarity to human NK cells – expressing all of the NCRs and secreting IFN-γ and TNF-α. These CD3^−^/NKp46^+^ cells from both healthy and OS-bearing canines exhibit robust expansion with K562 Clone9.mbIL21 and canine IL-2 and are highly cytotoxic against OS. In addition, we identified a small population of NKp46^−^ NK cells that have reduced cytotoxicity but similar cytokine secretion and can be induced to express NKp46.

This antibody also identifies a subset of T-cells that are NKp46^+^. In humans, NKp46^+^ T-cells are rare, but NKp46 can be acquired upon activation in γδ T-cells (45, 46). In bovines, NKp46^+^ T-cells have functional similarity to bovine NK cells as they were able to kill a tumor cell line spontaneously unlike T-cells that did not express NKp46 (47). We observed very little killing of the spontaneous OS lung metastasis cell line, Gray, by CD3^+^ canine T-cells, but it will be of interest to more fully characterize the phenotype and function of NKp46^+^ canine T-cells. Despite the low cytotoxicity of the T-cells against the OS cell line, it remains unknown whether the contaminating T-cells in K562 Clone9.mbIL-21 expanded cell cultures are capable of mediating graft-versus-host disease. Substantial work in humans suggests that contaminating T-cells might mediate GvHD and be detrimental in an allogeneic transplant setting (48, 49). Thus, to reduce this potential adverse effect by contaminating T-cells, we demonstrate that CD3-depleted PBMC can give rise to greater than 96% pure CD3^−^/NKp46^+^ expanded NK cells.

Spontaneous animal models of cancer for the study of NK cell therapies in a syngeneic setting have to date remained elusive due to difficulty in expanding NK cells in other species outside of human and the high cost of primate research. Instead, preclinical models of adoptive NK cell therapy have relied largely on infusing human NK cells into xenogeneic mouse models which lack a complete tumor microenvironment and an intact immune system that may influence the effectiveness of immune-based therapies (36, 50). We report here an improved expansion platform for canine NK cells on K562 Clone 9.mbIL-21 feeder cells obtaining a median fold expansion of over 20,000 in 3 weeks when supplemented with canine IL-2. To the best of our knowledge, this is the largest fold expansion of NK cells from any mammal outside of human and is significantly greater than previously reported expansions of NK-like cells in canine (20, 22). Previous expansions of canine CD3^−^ NK-like cells have reported an average fold expansion of 140-fold in 3 weeks using cytokine alone (IL-2 and IL-15), 233-fold expansion in 2 weeks with EL08-1D2 feeder cells following CD5 depletion, and 90-fold expansion with K562 feeder cells and soluble IL-2, IL-15, and IL-21 in 3 weeks (20, 21). It will be of future interest to determine if the improved expansion of canine NK cells with mbIL-21 over mbIL-15 is also due to an increase in telomere length as we have previously demonstrated for human NK cells (36).

Surprisingly, we found that CD3^−^/NKp46^+^ cells displayed cytotoxicity against the MDCK cells used as normal controls. MDCK can undergo tumorigenic transformation, which could induce the expression of activating ligands (44). In addition, the existence of only one Ly49 gene and no KIRs in the canine genome raises the question of whether the single Ly49 can bind to all dog leukocyte antigens (DLA) (51), which could increase NK cell killing in the allogeneic setting. It will be necessary to better define canine Ly49–DLA interactions in order to predict these responses.

Similar to Grondahl-Rosado et al., we found that NKp46 was not constitutively expressed on all CD3^−^/Granzyme B^+^ cells (17). In the present study, we added to this knowledge, by finding that these NKp46^−^, Null cells could be induced to express NKp46 and have a distinct function – displaying reduced cytotoxicity compared to NKp46^+^ NK cells. In contrast to Grondahl-Rosado et al., we found here that NKp46^−^ cells produce similar amounts of IFN-γ among all other cytokines tested. These differences may be explained by different cell stimulation as Grondahl-Rosado et al. measured IFN-γ stimulation after culturing NKp46^+^ and NKp46^−^ cells together with human IL-2, IL-12, and IL-15 while, in this paper, we stimulated NKp46^+^ and NKp46^−^ cells after sorting with canine IL-2 and K562 expressing membrane-bound IL-21 (Figure 5F) (17).

NKp46^−^ NK cells are not unique to canines but have been previously reported in both porcine and humans, where NKp46 expression also discriminates human NK cells with reduced cytotoxicity. In contrast to our data in canine, porcine NKp46 expression discriminate porcine NK cells with different IFN-γ secretion but not cytotoxicity (27, 52). Similar to porcine NKp46^−^ NK cells, NKp46 could be induced in canine NKp46^−^ cells; however, NKp46 has not been inducible in human NKp46^−^ cells (27). This may be due to differences in the regulation of NKp46 expression across species, where it is inducible in some species’ (porcine and canine) NK cells, but not in human, or NKp46 expression may require specific signals that have not been described yet (52).

We found striking phenotypic similarity between canine NKp46^−^ null cells and both primary and K562 Clone9.mbIL-21-expanded NKp46^−^ human NK cells (CD3^−^/CD56^+^). In humans, NKp46^−^ NK cells are predominantly CD56^dim^. Expanded NKp46^−^ cells in both species have reduced expression of NKp44, NKG2D, and CD16. Thus, we speculate that NKp46^−^ canine and human NK cells may represent similar subsets of NK cells in both species; however, additional functional and phenotypic characterizations of NKp46^−^ NK cells are necessary to elucidate this cell type to determine if they are homologous. Primary human NK cells also have reduced expression of NKp44 and CD16, suggesting that this population of NKp46^−^ NK cells is not an artifact of *ex vivo* expansion. There are several possible explanations for this cell type in humans that will be of interest to explore in future studies. CD56^dim^/CD16^−^/NKp46^−^ cells may represent an intermediate step in NK cell maturation between CD56^bright^/CD16^low/−^/NKp46^+^ and CD56^dim^/CD16^+^/NKp46^dim^ NK cells, or CD56^dim^/NKp46^−^/CD16^−^ cells may be an exhausted NK cell population that were formerly CD56^dim^/CD16^+^/NKp46^+^ cells and have downregulated CD16 and NKp46 because of senescence.

Contrary to human OS patients, we found no significant difference in circulating NK cells in canine OS compared to healthy canines (53). It will be of future interest to determine if these primary NK cells are functionally impaired, which would be facilitating tumor growth and has been found in several human cancers (54, 55). However, our data demonstrate that expanded NK cells from canine OS patients are active against OS, both primary and metastatic cell lines. This parallels findings in human OS where cytokine-activated NK cells from human OS patients have similar cytotoxicity against both OS cell lines and autologous patient-derived biopsies compared to healthy controls (56). Based on these findings, autologous NK cells may be effective in canine OS.

In summary, our study furthers the understanding of the canine NK cell phenotype and function. Furthermore, we describe the largest expansion of NK cells from another mammal besides humans, using K562 Clone 9.mbIL-21 feeder cells and canine IL-2. These mbIL-21 expanded NK cells are highly cytotoxic against OS cell lines, suggesting potential for immunotherapy of dogs with OS and potential for therapeutic use in other canine cancers such as lymphoma, leukemia, melanoma, and glioblastoma. This approach will allow for the study of NK cell therapy in an immune-intact, outbred, and spontaneous animal model. Many of these cancers progress rapidly in canines, allowing for rapid testing of NK cell therapies, which is of particular interest in rare and pediatric cancers such as OS where trial accrual is slow. These results support the development of canine NK cell trials to help prioritize the most promising treatment regimens for human clinical trials. Of particular interest is a trial of intratumoral injection of canine NK-like cells expanded on K562 Clone9.mbIL-21 feeder cells after radiation therapy in canine OS that is currently underway (UC Davis, Michael Kent & Bob Canter). The effectiveness of canine NK cells in combination with other agents, such as cytokines, chemotherapy, and antibodies, will be of particular interest.

## Author Contributions

JF, EB, YY, and AA-L performed the experiments. JF, SS, and DL designed the experiments. JF, AA-L, SS, and DL interpreted the data. JF, AA-L, YY, and DL drafted the manuscript. All the authors approved of the final version for publication.

## Conflict of Interest Statement

JF, SS, and DL report a financial interest in antibody Clone 48A with respect to licensing agreements. The other authors declare no conflict of interest.

## Abbreviations

GM-CSF, granulocyte-macrophage colony-stimulating factor; IFN-γ, interferon gamma; IL, interleukin; IQR, interquartile range; mbIL-21, membrane-bound IL-21; NCRs, natural cytotoxicity receptors; NK, natural killer; OS, osteosarcoma; PBMC, peripheral blood mononuclear cells; RBCs, red blood cells; rcIL-2, recombinant canine IL-2; TNF-α, tumor necrosis factor-alpha.
